# A Microbiome-Driven Approach to Combating Depression During the COVID-19 Pandemic

**DOI:** 10.3389/fnut.2021.672390

**Published:** 2021-08-24

**Authors:** Mahmoud A. Ghannoum, MaryKate Ford, Robert A. Bonomo, Ahmed Gamal, Thomas S. McCormick

**Affiliations:** ^1^Integrated Microbiome Core, Department of Dermatology, Case Western Reserve University, Cleveland, OH, United States; ^2^University Hospitals Cleveland Medical Center, Cleveland, OH, United States; ^3^BIOHM Health LLC, Cleveland, OH, United States; ^4^Louis Stokes Cleveland Department of Veterans Affairs Medical Center, Cleveland, OH, United States

**Keywords:** gut microbiome, depression, holistic approach, diet, probiotics, exercise, sleep, stress management

## Abstract

The significant stressors brought about and exacerbated by COVID-19 are associated with startling surges in mental health illnesses, specifically those related to depressive disorders. Given the huge impact of depression on society, and an incomplete understanding of impactful therapeutics, we have examined the current literature surrounding the microbiome and gut-brain axis to advance a potential complementary approach to address depression and depressive disorders that have increased during the COVID-19 pandemic. While we understand that the impact of the human gut microbiome on emotional health is a newly emerging field and more research needs to be conducted, the current evidence is extremely promising and suggests at least part of the answer to understanding depression in more depth may lie within the microbiome. As a result of these findings, we propose that a microbiome-based holistic approach, which involves carefully annotating the microbiome and potential modification through diet, probiotics, and lifestyle changes, may address depression. This paper's primary purpose is to shed light on the link between the gut microbiome and depression, including the gut-brain axis and propose a holistic approach to microbiome modification, with the ultimate goal of assisting individuals to manage their battle with depression through diet, probiotics, and lifestyle changes, in addition to offering a semblance of hope during these challenging times.

## Introduction

Within a 1-month period at the beginning of the COVID-19 pandemic, there was a reported 34.1% increase in prescriptions for anti-anxiety medications, an 18.6% increase in antidepressant prescriptions, and a 14.8% increase in common anti-insomnia drugs including prescribed anti-insomnia medications including eszopiclone, zolpidem and zaleplonin the United States (1). During such a short period of time, this steep rise hints at the magnitude of COVID-19's immediate and widespread effect on mental health. In a recent Single Care survey taken from 1,000 U.S. respondents, 59% reported that their mental health had been affected by COVID-19 (2). Additionally, a spokesperson for the Substance Abuse and Mental Health Services Administration (SAMHSA)-Disaster Distress Helpline reported a 338% increase in the call volume for individuals experiencing emotional distress from February 2020 to March 2020, an 891% increase compared to the call volume from March of 2019 (3). This alarming rate of mental health crises is an extreme concern for countries worldwide. Experts from the United Nations World Health Organization (WHO) have urged governments to put tribulations regarding the upsurge and severity of mental health illnesses at the “front and center” of their COVID-19 response (4). The suggestion by the United Nations is not trivial, as it is based on the startling statistics that show the devastating toll the pandemic is having on individuals all around the world.

Increasing levels of depressive disorders are especially concerning for mental health. Prior to the pandemic, Major Depressive Disorder (MDD) affected > 350,000,000 people and was a leading cause of disability, morbidity, and mortality worldwide (5–7). To further highlight the severity of depressive disorders, an even larger proportion of the population suffers from undiagnosed subclinical symptoms of depression (8). This subclinical disorder is especially problematic, as individuals who are diagnosed with depression or experience depressive symptoms are not the only ones who suffer from the resulting detrimental effects. Instead, this disorder also adversely affects society as a whole. Depression and depressive symptoms are associated with decreased physical health, increased unemployment, impaired social functioning, reduced productivity, and suicide, all of which can result in high economic costs to society and increased demand on the healthcare system (9, 10).

Despite the toll that depression has on both individuals and society, understanding and effectively treating depressive disorders is difficult. Current research addressing the diagnosis and treatment of depression and mood disorders is ongoing, but needs more time to develop a full understanding of the complexities involved in these disorders and to develop novel strategies to treat these disorders. Currently, clinical trials show that ~30–40% of depressed patients do not respond to their first-line antidepressant treatment (11, 12), up to 70% do not achieve complete remission of symptoms (13), and 20% never show a reduction in depressive symptoms even when accounting for multiple antidepressants and various methods of action (14–16). Interestingly, some promising results of electroconvulsive therapy (ECT) have been noted (17), however, this therapy is also not without controversy (18, 19).

Notably, more than half of individuals prescribed antidepressants experience side effects (20), which can be so severe that some must stop the treatment altogether. Thus, as with numerous other drug treatments, side effects have a considerable toll on efficacy and proper usage. The damage caused by depression, combined with an incomplete understanding of the depth of this disease highlights the need to examine new potential methods to address depression and better understand how it influences our bodies.

While discovering effective approaches to depressive disorders has always been a priority, we believe it is especially important today. This urgency was highlighted by the recent COVID19 pandemic, where increased stress and mental health conditions including increased anxiety and sleep disruption/disorders were elevated, especially in healthcare workers worldwide (21, 22). A survey among an Indian population showed that the prevalence of anxiety among respondents (*n* = 392) was 25.3% which was very high compared to the estimated levels in a Indian National Mental Health Survey (2015–2016) for the general population (23, 24). Similar other online surveys have been conducted targeting various international groups (25–28). Although, the validity of answers is a general problem of online surveys, similar observations reflect a potential major issue. Furthermore, such problems cannot be avoided in these challenging times. Such stressors include, but are not limited to, the following: (i) the uncertainty caused by the worldwide COVID-19 pandemic; (ii) feelings of fear and isolation; (iii) disrupted workplaces and schedules; (iv) unemployment; (v) changes to daily routines; (vi) anxiety about future events and decisions, and (vii) other yet unknown world events. These significant stressors, associated with the pandemic, were abruptly placed on top of regular stressors caused by everyday living with little or no warning.

To make matters worse, the increased level of acute and chronic stress caused by COVID-19 does not appear to be abating (21, 22, 26–31). People are still afraid that they or a loved one will contract the virus, worry about the efficacy and side effects of vaccination, there is still not the same level of physical human interaction, a lack of a sense of normalcy lingers with social distancing rules and masks. People are struggling with unemployment and parents were required to adjust to a new normal regarding educating (at home) school age children. These worries were highlighted under the concept of COVID Stress Syndrome, a newly proposed syndrome that is still under investigation (32–34).

Here, we propose a potential approach to address depression, which involves analysis of the microbiome and potential modification through diet, probiotics, and lifestyle changes. This article discusses gut and brain communication and the potential connection to stress and depression, the potential association between COVID-19 and the microbiome, the reported association between the microbiome and depression, and how the microbiome and depression may relate to diet, probiotics, and lifestyle habits. Lastly, we conclude by offering a potential approach to addressing depression through lifestyle modification including diet, exercise and potential microbiome modification. The overall goal is to examine evidence suggesting a connection of the microbiome to the brain, which implicates potential effects on depression and depressive disorders, in order to address how individuals may contain and end their battle with depression and navigate these difficult times.

## COVID-19 and Microbiome

Since the beginning of the COVID-19 pandemic extensive efforts have been made to combat this new virus. One of the main steps in studying any infectious agent is to know how it can infect human cells and consequently cause symptoms. In this regard, studies have found that COVID-19 targets exclusively the angiotensin-converting enzyme 2 (ACE2) receptor as an entry receptor into ACE2-expressing cells (35) which have been identified as the surface receptor for the spike proteins of acute respiratory syndrome coronavirus (SARS-CoV) (36), a first and essential step of viral infections of host cells (37). Moreover, the virus also uses serine protease TMPRSS2 for S protein priming that facilitates viral spread and pathogenesis (38, 39). Thus, in addition to the mode of transmission playing a major role (40), the expression of ACE2 on respiratory epithelia cells make it more plausible (39, 41).

In addition to the well-documented respiratory route of infection and subsequent consequences (respiratory sequalea) (42), ACE2 is expressed on other tissues especially the gastrointestinal tract (41). This may explain extra-pulmonary manifestations associated with COVID19 infection (43–52). Although respiratory syndrome is the main presentation of COVID19 infection, gastrointestinal (GI) symptoms have been reported in a number of COVID-19 cases (53–56). This concept has been further validated by data reported by Xiao et al., showing that 73 patients infected with COVID-19 tested positive for viral RNA in their stools (52). However, the epithelium lining the esophagus, stomach, duodenum, and rectum showed no significant damage on staining. Furthermore, continued viral shedding in stools even after negative nasal swabs has been reported by Xu et al., in a study that included 10 pediatric COVID-19 infection cases confirmed by real time reverse transcription PCR assay of viral RNA. Although, a limitation for this study is small sample size and there was no evidence of replication-competent virus in fecal swabs. In another study by Gu et al., examining the gut microbial diversity of 30 patients with COVID-19, a marked decrease in microbiome diversity was observed when compared to healthy controls. Although this is a modest number of observed events (*N* = 30) the overall observation that the gut microbiome of the COVID-19 group was dominated by *Streptococcus, Rothia, Veillonella, Erysipelatoclostridium*, and *Actinomyces* was notable. On the other hand, the abundance of the *Fusicatenibacter, Anaerostipes, Agathobacter*, unclassified *Lachnospiraceae*, and *E. hallii group* was reduced (57). This data agreed with results from Zuo et al., that showed an increase in opportunistic pathogens known to cause bacteremia including *Clostridium hathewayi, Actinomyces viscosus*, and *Bacteroides nordii* compared with controls, as well as depletion of *Faecalibacterium prausnitzii, Lachnospiraceae bacterium, Eubacterium rectale, Ruminococcus obeum*, and *Dorea formicigenerans* in COVID-19 patients receiving antibiotic therapy (58). However, again, the study had a limited sample size (*n* = 25). Importantly, this study showed that *Clostridium ramosum and C. hathewayi*, were the top bacteria positively associated with COVID-19 disease severity, which is supported by the observation that these microorganisms have been shown to strongly upregulate colonic expression of ACE2 in the murine gut (59). ACE2 not only plays a role in COVID-19 infection, but also plays an important role in regulating the gut microbiome (60) which in turn regulates the host immune response and gene expression (61). Additionally, a decrease in ACE2 expression was found to be associated with decreased tryptophan amino acid absorption in murine studies (60) which has also been linked to disturbances in the gut microbiome and consequently disruption of the gut-brain-axis using *in silico* analyses (62).

In a separate clinical trial designed to further understand the effect of COVID-19 infection on the gut microbiome, viral RNA extraction and shotgun metagenomics sequencing were performed on fecal samples of 15 patients hospitalized with COVID-19 (63). Analysis of the samples showed active viral infection signatures in the fecal samples even after clearance of the virus from respiratory samples for up to 6 days with a higher infectivity signature in samples that had greater abundances of the bacterial species *Collinsella aerofaciens, Collinsella tanakaei, Streptococcus infantis*, and *Morganella morganii*. Although this study involved a limited number of patients, these were emerging reports during the pandemic, and acquiring large sample numbers was unlikely to occur. Similarly, analysis of fecal samples from 30 patients with COVID-19 in Hong Kong showed increased proportions of opportunistic fungal pathogens such as *Candida albicans, Candida auris*, and *Aspergillus flavus* compared with controls, where detection of *Aspergillus* was found in 2 samples even after clearance of the virus from nasopharyngeal samples and resolution of respiratory symptoms (64). Yu et al., also reported that reduction in the *Bifidobacterium, Lactobacillus*, and *Eubacterium*, as well as significantly increased *Corynebacterium* of *Actinobacteria* and *Ruthenibacterium* of *Firmicutes* were observed in four COVID-19 patients who had decreased immune cells and refractory hypoxemia during hospitalization (65).

Collectively, the previous reports shed light on the potential correlation between COVID infection and microbiome disturbance, however, these reports have limitations that necessitate further investigations with larger sample sizes that can be used to approximate the general population.

Although there were reports of COVID-19 patients exhibiting GI symptoms, high rates of fever (>38.5°C), fatigue, shortness of breath and headache (56), a study by Lean et al., published during preparation of our manuscript, showed that patients with digestive symptoms experienced less severe disease (66) as defined by the need for mechanical ventilation, ICU admission or death, which is further supported by similar observation in other studies (55, 67, 68). Additionally, patients with digestive symptoms had a significantly longer time from onset to admission than patients without digestive symptoms (55, 68), necessitating a high level of caution in reaching an early diagnosis and setup of necessary infection control measures to control the transmission rate. This inconsistent observation reflects our poor understating of the nature of this novel virus and warrant further investigation.

Another clinical reported manifestation is COVID-19 induced neurological symptoms (51, 69, 70) including non-specific cognitive complaints, headache, numbness/tingling, dysgeusia, and anosmia (loss of smell) (71–74). These observations were thought to be due to ACE2 expression on neurons and glial cells of the brain (41) allowing the virus to directly reach the brain via the olfactory bulb following inhalation without the use of ACE2, however, this was strongly refuted by growing evidence that support these neurological findings are mainly attributed to the effects of hypoxia, coagulopathy, and multi-organ damage in severe infection, accompanied by virus-mediated inflammatory processes (74).

## Gut-Brain Axis

In the past, scientists believed that the brain was the central control for all processes. However, recent findings suggest otherwise. Numerous studies have shown that the microbiota, or the communities of bacteria and fungi that live in our gut, impact our brain and emotions. The communication between the gut microbiome and the brain is bidirectional and occurs through neural, inflammatory, and hormonal signaling pathways. To further expand upon this relationship, murine studies have shown that the gut microbiome is actively involved in processes linked to brain development, physiology, psychology, and behavior (75, 76). Specifically, murine models have demonstrated that the gut microbiome plays a critical role in the regulation of mood, anxiety, and pain (77, 78). In addition, the brain-gut axis has been shown to modulate the following: stress responsiveness (79, 80), prefrontal myelination (81), brain biochemistry (78), immune function, neurotransmission, and neurogenesis (80) using murine models and germ free animal studies.

As a result of the bidirectional interaction between the gut microbiome and the brain, each can send messages that impact the other. Cortisol, which is the primary stress hormone that dictates which functions are essential during a fight-or-flight situation (82), is an example of how the brain can send messages that impact the gut. Observations in animal studies showed that administration of dexamethasone mimicked in a dose dependent manner the effect of maternal separation in preterm male and female pups which showed enhanced plasma corticosterone, increased *in vivo* intestinal permeability, and induced bacterial translocation to liver and spleen (83). A similar observation was reported in other studies using rat models (84). Furthermore, germ free mice demonstrated plasma ACTH and corticosterone elevation in response to stress that was higher in comparison to specific pathogen free controls that was reversed by reconstitution with *Bifidobacterium infantis*, suggesting gut microbiota can affect the development of the hypothalamic–pituitary–adrenal stress response in mice (83, 85). In humans, patients with irritable bowel syndrome (IBS) were found to have increased adrenocorticotropic hormone and cortisol levels in response to corticotropin-releasing hormone infusion and elevated levels of the proinflammatory cytokines IL-6 compared to controls, an observation that suggest over activation of the hypothalamic-pituitary-adrenal axis (86). Although this study had a limited sample size (*n* = 151), this evidence suggests a communication between the brain and the gut.

On the other hand, an imbalance in the gut's microbes, as reviewed by Petersen and Round (87), is an example of how the gut can send messages that impact the brain. Microbial shifts in abundance and diversity (either increased or decreased) are often termed “dysbiosis” (87–90). In the present article, our reference to dysbiosis is to indicate a change from “baseline” microbiota for any given individual, not necessarily to indicate a pathological outcome. However, in general, decreased levels of diversity have been associated with pathological outcomes often identified as dysbiosis. When dysbiosis is observed, numerous targets are affected, such as the levels of dopamine and serotonin in the brain, both of which directly influence mood using germ free murine models (91–95). It is worth mentioning that dysbiosis is not the only factor that affects the level of these transmitters, however, this observation strongly suggests that microbiota may have a role in regulating these neurotransmitters. Such changes are associated with increased depression and anxiety and altered eating habits (96, 97). In summation, the bidirectional interaction between the gut microbiome and the brain highlights the relationship that exists between the two.

## Is There an Association Between the Microbiome and Depression?

Given the reported increase in stress, anxiety and depression with the COVID19 pandemic, examining potential factors influencing depression is in order. One such factor is the microbiome connection to these disorders. The connection between the gut microbiome and depression is demonstrated by the subsequent factors that affect, and are affected by, the gut microbiome's composition and the onset/development of depression. The interaction between the gut microbiome and the brain lays the foundation for the specific link between the gut microbiome and depression. Changes to the gut microbiota in major depressive disorder adversely affect many dimensions of the gut-brain axis, including the following: hyperactivation of the Hypothalamic-Pituitary-Adrenal (HPA) axis, disruption of neural circuits and neurotransmitter levels, excess production of proinflammatory cytokines in the immune system, and disruption of the intestinal barrier, as reviewed by Bastiaanssen et al. (98).

Similarly, the gut microbiome, as reviewed by Rea et al., plays a critical role in the development and function of the HPA axis, which controls one's stress response and is involved in depression (99). Clinical evidence suggests that depressive episodes are associated with dysregulation of the HPA axis (100), and the stabilization of the HPA axis is connected to a reduction in depressive symptoms (101, 102). Additionally, the gut microbiome can affect both neurotransmission and the production of various neurotransmitters, some of which are related to depression (96, 97, 103). The microbiota has also been shown to directly impact the function of the central nervous system (CNS) via neuronal activation of stress circuits (98). For example, murine studies indicate gut microbes can regulate stress response through the activation of the vagal pathways when *Citrobacter rodentium* and *Campylobacter jejuni* are administered orally (104, 105). Mechanisms underlying the effects of the microbiome in psychopathological disorders (e.g., anxiety and depression) suggest that pro-inflammatory cytokines could act as mediators to induce anxious behavior as shown in clinical studies (106). In this regard, various cytokine receptors have been found on the vagus nerve and other peripheral and spinal nerves that may signal inflammation to the brain, which could lead to depression and anxiety (107). Alternatively, in individuals with vagotomy, cytokines could enter the brain via the circumventricular organs, which facilitate communication between the central nervous system and peripheral blood, or via cytokine transporters at the blood-brain barrier where pro-inflammatory cytokines could gain access to the brain through these saturable transport systems. Another pathway that may participate in inducing depression involves activation of interleukin-1 receptors (positioned on perivascular macrophages and endothelial cells of small blood vessels in the brain) by circulating cytokines leading to local production of prostaglandin E2 as determined in rat studies (108, 109).

The direct link between the gut microbiome and depression has also been supported through various studies with differing perspectives and experimental approaches including reviews and primary behavioral studies in rats (110–113). It has been discovered that around 40–60% of anxious and depressed individuals report having an intestinal function disturbance, like Irritable Bowel Syndrome (114). Furthermore, transferring fecal gut microbiota from humans with depression into rodents has been shown to induce depression-like states in the animals, highlighting the causal role that the gut microbiome plays in the onset of Major Depressive Disorders (MDD) (113, 115, 116).

While the findings above highlight the link between the microbiome and depression, more evidence to support this relationship has been established. For example, scientists can distinguish between individuals with and without depression by analyzing their microbiome makeup (49). In fact, studies using the 16S ribosomal RNA sequencing method have observed abnormalities in the gut bacterial microbiomes of individuals with MDD (117, 118). Specifically, depressed patients are shown to have reduced microbiome abundance and alpha diversity. For example, Aizawa et al. found that the amount of *Bifidobacterium* and *Lactobacillus* in the feces of patients with major depression is typically less than that of healthy individuals (119). In comparison, a study conducted by Valles-Colomer et al. examined the microorganisms present in 1,000 individuals and discovered that depressed individuals were often more likely to have a reduced abundance of *Coprococcus* and *Dialister* when compared to their non-depressed counterparts (120).

Another study conducted by Yang et al. identified 3 bacteriophages, 47 bacterial species, and 50 fecal metabolites showing notable differences in abundance between patients with MDD and healthy control subjects. Patients with MDD were mainly characterized by increased abundance of the genus *Bacteroides* spp. and decreased levels of the genera *Blautia* and *Eubacterium*. Furthermore, using a discovery and validation set, the authors identified a biomarker panel that robustly discriminated between MDD and healthy individuals (49). Additionally, most studies that have examined patients with MDD have found an association between depression and “increased levels of the phylum *Actinobacteria*, order *Bacteroidales*, family *Enterobacteriaceae*, genus *Alistipes* and deceased family *Lachnospiraceae*, and genus *Faecalibacterium*” (121).

The plethora of evidence supporting the gut-brain axis and the established relationship between depression and the gut microbiome is indicative of potential new and more effective depression management. Based on the findings above, it is clear that the gut microbiome's makeup in individuals with depressive disorders are disrupted and lack the appropriate levels of beneficial microorganisms. Thus, we believe that encouraging the growth of such beneficial microorganisms and rebalancing the gut microbiome in individuals with MDD may be a promising step toward assisting individuals ease their depression *via* the gut-brain axis. Therefore, we propose a multifaceted approach to manage depression that involves rebalancing and maintaining the gut microbiome through diet, probiotics, and specific lifestyle changes.

## Diet and Depression

While acknowledging the complex and multidirectional nature of the relationship between diet and mental health, we propose that diet is an essential part of a balanced approach to combating depression. A large number of promising studies have examined the relationship between diet and depression. In particular, one study examined 16 eligible randomized controlled trials with outcome data for more than 45,000 participants. The findings from this study indicate that dietary intervention significantly reduced depressive symptoms in high-quality trials (g = 0.321, 95% CI = 0.12–0.53, *p* = 0.002) (122). Other meta analyses and clinical studies have revealed an association between diet quality and the probability of and risk for depression (123, 124). Additionally, pro-inflammatory dietary patterns have specifically been linked to a greater likelihood of developing depression and depressive symptoms in clinical studies (125–127).

Eating patterns during the COVID pandemic showed an increased adoption of unhealthy diets. A study by Robinson et al., examining the impact of COVID-19 on a large sample of UK adults (*n* = 2,002) during social lockdown showed that 82% reported an increase in the intake of unhealthy food (128). Furthermore, 36% of the participants reported an increase in alcohol intake compared to before lockdown which is known to cause disruption of the gut microbiome (129) by causing bacterial overgrowth (130, 131) and hyper-permeability of the intestinal membrane (i.e., leaky gut) (132). In this regards, Pollard et al., also reported an increase in alcohol intake of 14% over the baseline in 2019 during the COVID-19 pandemic in a sample that included 1,540 adults in the U.S. (133).

Studies have also established a relationship between the prevalence of depressive symptoms and certain eating habits. For example, depressive symptoms have been associated with a high glycemic index attributed to a high intake of sugars and refined carbohydrates (126, 127, 134). Conversely, healthy eating habits that limit sugar intake and refined carbohydrates are associated with a decreased risk of depression. Such findings align with several smaller and more recent trials where considerable improvements in depression levels were observed in people who followed the so-called “Mediterranean Diet” (135–137). For context, the Mediterranean diet, which adheres to healthy eating practices, is associated with a small intake of red meat; a high intake of vegetables, fruits, beans, and nuts; and a medium intake of eggs, dairy, and poultry (123, 126, 127).

We propose that the consistently significant and positive effects of dietary intervention on depressive symptoms reflect the impact that diet has on the microbiome. While recognizing the role that genetics and antibiotics play, we believe that diet is a potentially adjustable determining factor of the relative abundance, diversity, and functionality of organisms comprising the gut microbiome. In the same vein, no single treatment is likely to fully ameliorate depression and/or depressive symptoms, in fact, combined therapeutic approaches (a holistic approach) may include several areas including pharmacological interventions, psychiatric therapy and lifestyle and nutritional changes. For example, studies have shown that the artificial sweetener Splenda™ promotes gut dysbiosis and the increased growth of *Proteobacteria*, which is an indicator for inflammation (138). Additionally, decreased function of the gut barrier, also known as “leaky gut,” is linked to an “unhealthy” gut microbiome that is attributed to a diet high in saturated fats, refined sugars, artificial sweeteners, and low in fiber, aka the “Western” diet (125, 139, 140). On the other hand, a diet high in fibers, polyphenols (flavonoids and phenolic acid micronutrients found in berries, nuts, flaxseeds, vegetables, olives, coffee, and tea) and unsaturated fatty acids (fatty acids that have one or more carbon-carbon double bonds, comprised of monounsaturated fatty acids present in avocado, nuts, olive, peanut oils and vegetable oils, and polyunsaturated fatty acids present mainly in vegetable oils such as sunflower, sesame, soybean, and corn oils, the prototypical “Mediterranean” diet) is associated with improving and promoting the growth of beneficial gut microbial taxa (139, 141).

Although the exact underlying mechanisms through which diet impacts mental health have yet to be defined, there are several pathways by which diet can play a role. These pathways are related to oxidative stress, inflammation, and mitochondrial dysfunction, which tend to be disrupted in people with mental health disorders (142). A healthy diet typically includes various bioactive compounds that beneficially engage these pathways. For example, fruits and vegetables contain fiber, vitamins, minerals, and are packed with a large number of polyphenols. These nutritional factors appear to be associated with decreased depression rates, possibly due to their anti-inflammatory, neuroprotective, and prebiotic properties (143, 144). On the other hand, unhealthy diets are rich in nutritional factors that may negatively engage these pathways. Specifically, elements commonly found in processed foods like artificial sweeteners, emulsifiers, and saturated fatty acids may change the gut microbiome's composition and activate inflammatory pathways (136).

## Probiotics and Depression

In addition to diet, we propose that probiotics are an essential part of an encompassing approach to treating depression. We assert that probiotics will help to serve the critical function of rebalancing the microbiome. While we recognize that the study of probiotics in humans is a newly emerging field that has recently gained an immense amount of attention, published evidence suggests that probiotics will play a promising role in combating depression (145). Numerous studies conducted on both humans and animals have indicated that probiotics are associated with a reduction in anxiety and depression (78, 80, 146–149). Other clinical studies have shown that probiotics have also effectively mitigated anxiety and depressive symptoms in a manner that is comparable to that of conventional prescription medications (150–152). Additionally, related studies have indicated that probiotic use reduced depressive symptoms and improved the functionality of both the HPA axis and was equivalent to treatment with the antidepressant Citalopram in murine studies (106, 146, 152). However, as with any emerging therapeutic option, some failed to achieve the desired effect, a result that has been reported in a number of studies (153–155).

One particular study gave patients suffering from chronic stress a 3-week probiotic regimen that contained *Lactobacillus casei*. Subjects in the bottom third of the elated/depressed scale demonstrated the most improvement as they consistently rated an overall happier mood (150). Additionally, a double-blind, placebo-controlled randomized study examined the effects of probiotics on behavior, brain function, and gut microbial composition in 45 healthy volunteers. At the end of the 4-week period, probiotic administration was associated with shifts in gut microbiome profiles and brain activation patterns. The probiotic arm in this study contained the following nine bacterial strains: *Lactobacillus casei* W56, *Lactobacillus acidophilus* W22, *Lactobacillus paracasei* W20, *Bifidobacterium lactis* W51, *Lactobacillus salivarius* W24, *Lactococcus lactis* W19, *Bifidobacterium lactis* W52, *Lactobacillus plantarum* W62 and *Bifidobacterium bifidum* W23 (156). Using probiotics that incorporate multiple strains may provide better benefits than those using single strains and may result in having synergistic effects that can substantially enhance the establishment and competitiveness of a putative probiotic strain in the GI tract. In this regard, a review by Sun and Buys showed that consumption of probiotics that have multiple strains in combination as well as of probiotics containing *Lactobacillus acidophilus*, a mixture of *Lactobacillus acidophilus* and *Bifidobacterium lactis*, or *Lactobacillus plantarum* led to a statistically significant reductions in total and low-density lipoprotein (LDL) cholesterol levels, while clinical trials that used a single strain did not (157). We speculate that each strain may contribute differently (e.g., some produce more beneficial short chain fatty acids (SCFA), while others antagonize pathogenic microorganisms present in the gut).

In addition to the strains previously mentioned, *Bifidobacterium infantis* (146), *Lactobacillus helveticus* (106, 147), *Lactobacillus rhamnosus* (78), and *Bifidobacterium longum* (137) have also been associated with a reduction in depression. Studies have suggested that probiotics with such bacterial strains modify the microbiome and potentially treat depression due to their ability to normalize cortisol levels, regulate the HPA axis, and reduce circulating pro-inflammatory cytokines (96, 106).

Potential disruption of gut microbiome caused by COVID-19 result in colonization of the gut with opportunistic pathogens, increasing the risk of biofilm formation that was shown to be associated with many GI diseases (158, 159) that are resistant to standard treatment. In a recent study a probiotic combination of *Saccharomyces boulardii, Lactobacillus acidophilus, Bifidobacterium breve*, and *L. rhamnosus* was shown to provide anti-biofilm activity *in vitro* against polymicrobial biofilms formed by *C. albicans* or *C. tropicalis* when combined with *Escherichia coli* and *Serratia marcescens* (160). Thus, use of probiotics may help in restoring gut microbiome balance, as well as, reducing the risk of biofilm formation.

The underlying mechanism/s for the contribution of probiotics to rebalance the microbiome, normalize neuroendocrine system, and reduce depression may be mediated by: (1) immunomodulatory effects of the probiotics, (2) ability to decrease intestinal translocation of microbial constituents across the gut intestinal barrier, and (3) inhibition of digestive biofilms. However, the studies that suggest these potential influences are mainly associative, with little mechanistic considerations. The potential involvement of immunomodulatory effects of probiotics (e.g., *B. pseudocatenulatum*) is indicated by a pronounced decrease of stress-induced interleukin (IL)-18 levels in the serum as well as decrease of basal and stress-induced interferon gamma (IFNγ) levels in the small intestine of probiotic-treated animals. Additionally, probiotic incorporation led to a reduction of corticosterone in stool and catecholamines in the hypothalamus, as well as anxiety-like behavior (161). Such findings indicate that probiotic therapies may produce certain benefits over current therapeutic drugs or play an adjunctive role. As a result of this, we propose that probiotics may play an important part of a comprehensive approach to rebalancing the microbiome and treating depression.

## Lifestyle Habits and Depression

The last element proposed for combating depression focuses on 3 specific lifestyle habits; exercise, sleep, and stress management. The gut microbiota is an extremely dynamic system that constantly changes over time due to a combination of various factors and lifestyle habits. Since the beginning of COVID-19 pandemic, the fear of being infected, loss of loved ones, lockdown, and social isolation all together caused marked changes in individuals' behaviors in an attempt to combat the stress caused by these factors. This in turn caused a huge impact on daily activities in multiple aspects including physical activity (162), eating habits (128), and sleeping (163). We propose that these three lifestyle habits are critical as a result of the existing evidence that demonstrates their unique benefits in improving gut health and decreasing depression.

### Exercise and Depression

The first lifestyle habit addressed is exercise. Studies showed that COVID-19 negatively affected daily physical activity, thereby shifting individuals to more sedentary lifestyle (164). Zheng et al., investigated the impact of the pandemic on physical activity in a sample of 631 young adults. In total, 70% of the participants reported decreased physical activity levels since the onset of the COVID-19 pandemic with a significant increase in sleep duration and sedentary behavior (e.g., computer/video games, sitting talking on telephone, doing computer/paper work), consistent with data reported by Moore et al., demonstrating significant decline in all types of physical activity among children and adolescents in Canada since the beginning of the pandemic (164). A recent review has shown that regular exercise plays an important role in increasing the diversity of the gut microbiota and maintaining the balance between beneficial and pathogenic bacterial communities (165). Consequently, this major decline in physical activity eventually causes significant disruption to the gut microbiota (i.e., dysbiosis) which in turn plays a role in multiple neuropsychiatric disorders (e.g., depression), as reviewed by Belizario and Faintuch (166).

We propose exercise as a significant lifestyle habit to consider due to its relationship with the microbiome and depression. Exercising is associated with various benefits that can be directly or indirectly related to microbiome health. For example, studies have indicated that many conditions involving inflammation, like depression, can be treated through exercise (167). Additionally, in clinical studies, exercise has been shown to reduce depressive symptoms (168–171) and the effects of stress-induced intestinal barrier dysfunction has been demonstrated using murine models (172).

The underlying mechanism/s responsible for the reduction in depression symptoms associated with exercise is unclear. However, a number of mechanisms have been proposed including the thermogenic, endorphin, and the monoamine hypothesis. Animal studies provide supportive evidence to the latter hypothesis where an increase in the availability of the brain neurotransmitters dopamine (a feel-good endorphin), norepinephrine, and serotonin have been observed in plasma, urine and various brain regions following exercise (168). Recent rodent studies continue to suggest that exercise may alleviate depression through common neuro-molecular mechanisms (173, 174), including increased expression of neurotrophic factors [i.e., Brain-derived neurotropic factor (BDNF)], regulation of HPA-axis activity (175), increased availability of serotonin and norepinephrine (176), and reduced systemic inflammatory signaling (177). These activities stimulate the development of new neurons, increase cerebral vasculature in human as well as animal studies (178, 179) and synaptic connections between neurons thereby boosting cognitive function with the ultimate result of reducing depression symptoms.

While the exact reasons are unknown, the evidence is abundantly clear that exercise helps to decrease depression and depressive symptoms. As a result of these findings, we propose that exercise is a critical lifestyle element to consider when dealing with depression. Specifically, performing a minimum of 150 min of moderate exercise every week, consistent with the U.S. government's Physical Activity Guidelines for Americans (180) and the American Heart Association's recommendations, may improve depressive symptoms (181).

### Sleep and Depression

In addition to exercise, sleep is another essential lifestyle element to consider, as it is also associated with the microbiome and depression. When it comes to sleep and the microbiome, sleep has been shown to have a direct impact on the microbiome in many ways. For example, murine studies have demonstrated the link between sleep deprivation or disruption and microbial dysbiosis (182, 183).

Gualano et al., investigated the effects of Covid-19 lockdown on mental health and sleep disturbances in a clinical study in Italy and showed an increase in the prevalence of depression and anxiety symptoms and sleep disturbances during the Covid-19 lockdown compared to before the lockdown 24.7 and 23.2% of the participants reporting depression and anxiety symptoms, respectively; while 42.2% had sleep disturbances (163). This was in agreement with other studies that assessed the impact of COVID-19 lockdown on mental health in Italy and China (21, 30, 184–186).

Lack of sleep may lead to and exacerbate depression and depressive symptoms. In fact, numerous studies have shown that individuals with depression experience trouble falling or staying asleep (187, 188), and individuals who have sleeping problems are more likely to become depressed in the future (189–192). These findings highlight the critical role sleep plays in depression and its impact on the microbiome. As a result of this, we propose that obtaining adequate sleep is a vital lifestyle element to consider when treating depression. Specifically, adults should attempt to receive 7–9 h of sleep per night, as suggested by the National Sleep Foundation's recommendations (193).

The relationship between sleep and the microbiome is bi-directional where the microbiome can affect our sleep pattern and sleep can impact the microbiome composition. Smith et al. (194) investigated the associations between sleep and the microbiome in human clinical studies and showed that microbiome diversity (richness and Shannon diversity indices) was positively correlated with sleep efficiency, and total sleep time, while negatively correlated with sleep fragmentation (characterized by repetitive short interruption of sleep). These findings suggest that gut microbiome diversity promotes healthier sleep. Specifically, richness within the phyla *Firmicutes* and *Bacteroidetes* were positively correlated with sleep efficiency. Other authors showed that in humans these two phyla are associated with sleep quality, circadian rhythm (internal process that regulates the sleep-wake cycle) and food intake, factors known to influence sleep quality (195, 196). Unlike *Firmicutes* and *Bacteroidetes* phyla, richness within the *Actinobacteria* phylum was shown to negatively correlate with the number of awakenings. In other words, increased richness within this phylum contributes to high sleep quality. The positive effect of the identified phyla on sleep could be attributed to their ability to secret the metabolite γ-aminobutyric acid, a neurotransmitter known to promote sleep (197).

The effect of sleep on the gut microbiome has also been investigated. Studies by Li et al. showed that a shift in circadian rhythms can elicit a change in the intestinal microbiota (198). While a publication by Benedict et al. showed that after 2 days of partial sleep deprivation vs. after 2 days of normal sleep, subjects had a significant increase in *Firmicutes*:*Bacteroidetes* (F/B) ratio, a measure thought to influence the maintenance of normal intestinal homeostasis. An increase or decrease in F/B ratio is often regarded as dysbiosis, however newer studies also question the reproducibility of this ratio (199). Furthermore, 2 days partial sleep deprivation resulted in an increase in the abundance of *Coriobacteriaceae* and *Erysipelotrichaceae* families, and reduced levels of *Tenericutes* (all *P* < 0.05). However, partial sleep deprivation did not have any effect of ß-diversity. These findings suggest that sleep deprivation in human subjects can induce alterations in bacterial families residing in the gut (200).

Interestingly, Li et al. (198) suggested that a bidirectional connection between the gut microbiome, sleep and depression exists with inflammation and endocrine hormones contributing to this process. In this regard, sleep loss, prolonged disturbances of circadian rhythms, and depression affects the metabolism of commensal gut bacteria and leads to alterations in microbiome structure including a reduction in members of the *Lactobacillaceae* family and an increase in *Enterococci, Bacteroides multiforme, Lachnospiraceae*, and *Ruminococcaceae*. These changes lead to microbiome dysbiosis (201–203). Damage to the gut epithelial barrier, as happens in leaky gut, will allow bacterial cells and their harmful metabolites to stimulate inflammatory immune responses thereby exciting the vagus and spinal afferent nerves (204). It has been postulated that the underlying mechanism for this process may be that the inflammatory response brought about by gut microbiome dysbiosis exacerbates insomnia and depression (112, 205–208).

### Stress and Depression

The last major lifestyle habit that comprises a complete approach to combating depression is effective stress management. We propose that stress management is an important lifestyle habit mainly because stress heavily impacts the microbiome and is related to depression. As a result of the gut-brain axis, there is always a link to the gut when a stress response occurs (97). For this reason, stress has been shown to have a significantly negative impact on the microbiome as it affects intestinal barrier function (209) and host-microbe interactions (210). Studies have demonstrated that various types of stressors, including the burden of COVID-19 pandemic (211–213), heat, social rejection, and separation, are responsible for compositional alterations to the gut microbiome (207, 214–218). In this regard, a murine study by Galley et al., showed that colonic lumen tissues were differentially affected by exposure to the psychological stressor imposition of restraint. Specifically, restraint stress significantly reduced the alpha diversity in the microbiome of stressed mice compared to controls. Moreover, a significant reduction in the relative abundance of the genera *Lactobacillus* and *Adlercreutzia* was observed (219).

In response to stress, the HPA axis produces cortisol to help the body deal with danger (220). This type of stress response relates to an array of neurological issues, including depression. In fact, studies have shown that the hyperactivity of the HPA axis is one of the most common neurobiological changes in patients suffering from depression (221, 222). Additional research has also demonstrated a “robust and causal” relationship between stressful life events and depression (223). Furthermore, a gastrointestinal connection exists as stress is a top risk factor for IBS, which is often linked to depression as outlined in recent review articles (224–226).

As such, we propose that effective stress management is an essential lifestyle habit to explore when combating depression. Specifically, the following stress management strategies, which include, but are not limited to, the following: taking time for hobbies, fostering healthy friendships, volunteering, seeking counseling when needed, and other relaxation techniques like yoga, meditation, deep breathing, and massage treatment.

While we understand that various lifestyle habits can positively impact depression, treatment should also include psychological, neuropsychiatric and pharmacological approaches. We propose that exercise, sleep, and stress management are among the most accessible methods to individuals that can be relatively easily implemented. Given the various research studies that have linked the microbiome and depression with these three particular lifestyle habits, we propose that this approach may hold promise.

## Proposed Approach to Rebalance the Microbiome and Reduce Depression

Having highlighted a relationship between depression and the microbiome, and the given association of COVID19 with stress and depression, we propose that one approach to combat depression should include potential diet modification, nutritional supplements and lifestyle changes. Specifically, increasing the abundance of beneficial organisms that exhibit a reduction in their levels during stress and depression (e.g., *F*. *prausnitzii, Bifidobacterium*, and *Lactobacillus*) and decrease the levels of pathogens such as *Candida, Corynebacterium* and *Ruthenibacterium* should provide an improved gut microbiome.

### A Prototypic Diet

Healthy eating habits involving limiting consumption of sugar and refined carbohydrates and are associated with a decreased risk of depression. One such diet, the Total Gut Balance (TGB) (97) incorporates all aspects of the microbiome using foods and lifestyle practices to support a healthy gut and reduce overall inflammation with potent anti-inflammatory and antioxidant foods rich in targeted vitamins, minerals, and other beneficial constituents. This diet is nutritionally balanced, whole-food based, low- glycemic rich in fiber and resistant starches, low in sugar, full of mono- and polyunsaturated plant fats, low in saturated fat and animal fat, incorporating lean plant protein and animal protein primarily from seafood, with some poultry, as outlined in Table 1. As shown, this diet closely resembles the “Mediterranean” diet in the makeup of the constituents (227).

**Table 1:** Overview of the total gut balance diet.

**Frequency**	**Food/food type**	**Quantity**
Daily	• Coconut or extra-virgin olive oil • Resistant starch foods • Cruciferous vegetables • Mycobiome-friendly vegetables • Apple cider vinegar OPTIONAL • Eggs • Poultry (EX: chicken and turkey) • Low-fat or non-fat dairy products • Tofu and tempeh • Edamame	• At least 1 tablespoon • At least 1 cup but no more than 2 cups • At least 1 cup with no maximum • At least 2 cups • At least 1 tablespoon • 2 • 6 ounces • 1 cup • 4–6 ounces • 1 cup, 2 servings maximum
3–7 times per week	• Fish/seafood • Ground turmeric • Ginger • Garlic • Pistachios and/or walnuts • Green Tea • Fermented Foods	• Up to 6 ounces • At least 1 teaspoon • At least 1 teaspoon dried and ground or 1 tablespoon fresh grated • 1 to 2 cloves • ¼ cup • At least 1 cup • ½ cup
Limited consumption	• Alcohol • Maple syrup or raw honey • Coffee and/or black tea	• No more than 3x/week or exclude completely • No more than 1 tablespoon per day, less is better • Prioritize organic varieties
Never	• No added sugar sweeteners (except for maple syrup and raw honey) • No refined grains • No processed, cured meat • No processed or packaged food with more than three ingredients • No oils or fats, other than those allowed above, including butter • No full-fat dairy products	

Adherence to the TGB diet was evaluated in a small clinical study of 10 individuals (228) where it was noted that the TGB diet counteracts microbiome imbalance.

Overabundance of Proteobacteria in the GI tract reflects microbiome imbalance and an unstable gut microbiome structure that could lead to inflammatory symptoms. Moreover, previous animal studies suggest that the microbial dysbiosis in IBD is associated with an increase in the levels of pathogenic Proteobacterial taxa such as *Enterobacteriaceae* (e.g., *E. coli*) and a reduction in protective *Firmicutes* species such as *F. prausnitzii* (229).

Individuals participating in the TGB exhibited an increase in *F. prausnitzii*, an organism reduced in depressed subjects. In contrast, high levels of *F. prausnitzii* are found in healthy subjects (230). *F. prausnitzii* is a butyrate producer that has shown anti-inflammatory effects using *in vitro* and *in vivo* mouse colitis models. Finally, a decrease in the abundance of pathogenic *Candida* spp. was also observed in TGB participants.

The observed increase in the level of *Roseburia*, a Gram-positive anaerobic bacterium, may have a positive impact on the microbiome of depressed individuals. *Roseburia* is a commensal bacterium that produces short chain fatty acids (SCFA), especially butyrate, which are anti-inflammatory and shown to help maintain immunity (231).

In addition to the described shift in the bacterial and fungal gut communities of TGB participants, an increase in abundance of beneficial fungi and bacteria and a corresponding decrease in harmful microorganisms, a shift associated with reported improved GI symptoms, less fatigue, more energy, better sleep, and fewer cravings for empty-calorie foods was also reported (228).

Studies have shown that bacteria inhabiting our gut have the ability to produce and/or consume a wide range of mammalian neurotransmitters, including dopamine, norepinephrine, serotonin, or gamma-aminobutyric acid (GABA) (91–95, 232). Since it is well-known that disturbance of these neurotransmitters is linked to a variety of mental disorders including depression (233–237), a target of many antidepressant pharmacological treatments, a possible approach to rebalance these neurotransmitters, although not completely discovered, is by modulating the abundance of these bacteria. However, some gut-derived neuro transmitters function differently from brain-derived neurotransmitters (238). It has been reported that *Bifidobacterium* improved the expression of Tph1 and secretion of 5-hydroxytryptophan (5-HTP) in RIN14B cells, significantly reduced depressive behaviors of mice, and increased the level of 5-hydroxytryptamine and brain-derived neurotrophic factor concentration in brain (239). Furthermore, in some studies, autistic patients were found to have increased level of *Clostridia* that were associated with production of toxic compounds, such as 3-hydroxypropionic acid (240) and p-cresol (241), found to inhibit the dopamine beta-hydroxylase that converts dopamine to norepinephrine in neurons in the brain and in the sympathetic nervous system (242). This increase in dopamine in neurons leads to production of oxidative species, depletion of glutathione and subsequently brain damage (240, 243, 244). Thus, increasing these beneficial organisms while decreasing pathogenic ones through diet may help in rebalancing neurotransmitter and reducing symptoms of some mental disorders including depression. However, investigations in this regard is still ongoing and thus can't be used as a solo treatment option.

### Probiotics

Based on association between COVID-19 infection and gut microbiome disruption, a logical approach to help balance the gut microbiome is administration of probiotics. Probiotic, as defined by World Health Organization, is “live microorganisms which when administered in adequate amounts confer a health benefit on the host” (245, 246). Not only should probiotic consumption restore the gut balance, it may also decrease the likelihood of colonization of the gut by opportunistic pathogens (247), as reported in many studies that analyzed the gut microbiome in COVID-19 infected patients (57, 58). Published reports have shown that use of a probiotic helped in supporting the immune system (248), as well as leading to a marked reduction in the incidence of respiratory tract infection (249) Furthermore, studies have also shown that lactic acid bacteria probiotics possess antiviral properties (250) which has been used in many fields including agriculture (251), poultry (252), and medicine (253).

### Lifestyle Recommendations

Eating to enhance the microbiome may help to achieve better health, but there is compelling research to show that other aspects of life, from the environment you live in, as well as sleep, stress, and exercise, that can significantly impact all gut microbes. While genetics is certainly part of this equation, and diet is another component, research continues to demonstrate that lifestyle factors play an important role in chronic diseases (254, 255). Because depression has been shown to be linked to dysbiosis in the microbiome, knowing what you can do to make your lifestyle habits work for you instead of against you is also important. To aid this approach, we propose a number of lifestyle recommendations that may enhance the microbiome balance which should result in reduced depression. A summation of the recommended lifestyle targets is listed in Table 2.

**Table 2:** Lifestyle recommendations.

**Lifestyle**	**Recommendation**
Exercise	Engage in a minimum of 150 min of moderate exercise per week, including aerobic exercise, strength training, and some stretching.
Sleep	Sleep for 7–9 h each night. To discover your specific needs, listen to your body to determine what amount of sleep makes you feel the best. You should get enough so that you feel refreshed when you wake up and you don't get too tired during the middle of the day, but you shouldn't sleep so much that you have a hard time getting moving.
Smoking	Don't do it. If you need help quitting, talk to your doctor. There are many effective Therapies.
Nutrient	Get a wide range of nutrients from a diverse diet by eating many different items on the Mycobiome Diet food lists. The more variety of healthful whole foods you eat— especially vegetables and fruits— the more diverse and robust your microbiome will be.
Drug	If you don't need a medication and your doctor agrees, don't take it.
Stress	Actively managing your stress is one of the best things you can do for your microbiome. Work purposefully to manage your stress for better life quality as well as better health and microbiome balance (with all its many benefits), via the gut– brain axis. Try to practice at least one stress management technique every day.

## Conclusion

As a result of the toll COVID-19 has had on mental health, and society's imperfect understanding and inability to adequately treat depression, we propose it is important to examine more thoroughly new potential methods to prevent and treat depression. While this topic still needs further examination, research is extremely promising and should offer a glimpse of hope. The overall goal of the present manuscript is to offer suggestions that incorporate an encompassing approach to combat depression. As such, we want to emphasize the importance of rebalancing the gut through probiotics, diet, and lifestyle changes. However, we also propose that more can and should be done to achieve this goal and improve the lives of individuals who are struggling.

Regardless of whether people have seen a decrease in their own mental well-being since the start of COVID-19, everyone needs to be responsive and work together to effectively bring an end to this mental health crisis. We propose that the first step toward progress is awareness. However, each of us cope with increased stressors in our own unique ways, such as remaining busy, spending time with family and friends, etc. It is critical to note that some community members are overwhelmed and are desperately in need of assistance. For these reasons, we recommend checking in on your loved ones and asking how you might be able to assist them during these unprecedented times.

Since the start of time, humans have continued to evolve and adapt to whatever situations they are presented with. We propose that the COVID-19 pandemic is no different from these situations. Therefore, if you find yourself constantly worrying about the future, we hope this paper has eased your concerns and allowed you to adopt our optimistic view that we will get through this and be better because of it.

## Data Availability Statement

The raw data supporting the conclusions of this article will be made available by the authors, without undue reservation.

## Ethics Statement

Ethical review and approval was not required for the study on human participants in accordance with the local legislation and institutional requirements. The patients/participants provided their written informed consent to participate in this study.

## Author Contributions

MG: conceptualization and funding acquisition. MF and MG: literature acquisition and writing—original draft. MG, TM, RB, and AG: writing—review and editing. All authors contributed to the article and approved the submitted version.

## Conflict of Interest

MF was an employee at BIOHM Health LLC. MG was the Co-founder of BIOHM Health LLC and author of the Total Gut Balance Book. The remaining authors declare that the research was conducted in the absence of any commercial or financial relationships that could be construed as a potential conflict of interest.

## Publisher's Note

All claims expressed in this article are solely those of the authors and do not necessarily represent those of their affiliated organizations, or those of the publisher, the editors and the reviewers. Any product that may be evaluated in this article, or claim that may be made by its manufacturer, is not guaranteed or endorsed by the publisher.
